# Cellular Chitchatting: Exploring the Role of Exosomes as Cardiovascular Risk Factors

**DOI:** 10.3389/fcell.2022.860005

**Published:** 2022-03-31

**Authors:** Giulia Germena, Laura Cecilia Zelarayán, Rabea Hinkel

**Affiliations:** ^1^ Laboratory Animal Science Unit, Leibniz-Institut für Primatenforschung, Deutsches Primatenzentrum GmbH, Göttingen, Germany; ^2^ DZHK (German Center for Cardiovascular Research), Partner Site Göttingen, Göttingen, Germany; ^3^ Institute of Pharmacology and Toxicology, University Medical Center Göttingen, Göttingen, Germany; ^4^ Institute for Animal Hygiene, Animal Welfare and Farm Animal Behaviour (ITTN), Stiftung Tierärztliche Hochschule Hannover, University of Veterinary Medicine, Hannover, Germany

**Keywords:** exosomes, diabetes, exercice, atherosclerosis, cardiovascular disease

## Abstract

Exosomes are small bi-lipid membranous vesicles (30–150 nm) containing different biological material such as proteins, lipids and nucleic acid. These small vesicles, inducing a cell to cell signaling pathway, are able to mediate multidirectional crosstalk to maintain homeostasis or modulate disease processes. With their various contents, exosomes sort and transfer specific information from their origin to a recipient cell, from a tissue or organ in the close proximity or at distance, generating an intra-inter tissue or organ communication. In the last decade exosomes have been identified in multiple organs and fluids under different pathological conditions. In particular, while the content and the abundance of exosome is now a diagnostic marker for cardiovascular diseases, their role in context-specific physiological and pathophysiological conditions in the cardiovascular system remains largely unknown. We summarize here the current knowledge on the role of exosomes as mediators of cardiovascular diseases in several pathophysiological conditions such as atherosclerosis and diabetes. In addition, we describe evidence of intercellular connection among multiple cell type (cardiac, vasculature, immune cells) as well as the challenge of their *in vivo* analysis.

## Introduction

Extracellular vesicles (EVs), with their size range of 30–2000 nm in diameter, are a heterogeneous group of secreted bilayer lipid particles originated from the endocytic pathway (Raposo and Stoorvogel, 2013).

In response to stimulation, EVs are secreted in the extracellular milieu by several cell types. They can transport a variety of substances including proteins, mRNAs, miRNAs, DNA and lipids, that can act as autocrine or paracrine factors but can also be shuttled to other cell types. EVs are classified as exosomes (Exos), microvesicles (MVs) and apoptotic bodies on the basis of their biogenesis and size. EVs are extensively described elsewhere (Gurung et al., 2021) and for the purpose of this review, only a general description is provided.

Within the extracellular vesicles groups, exosomes are 30–300 nm heterogeneous vesicles generated by the endocytic pathway in a three-step process including: 1-intracellular invagination of endosomes generating intraluminal vesicles (ILVs), 2-formation of multivesicular bodies (MVBs) and 3-released by fusion with the plasma membrane (Kowal et al., 2014).

MVBs (or late endosomes) are formed by the maturation of early endosomes and their formation involves the activation of the endosomal sorting complex required for transport (ESCRT) machinery, that drives membrane budding by recognizing and sequestering ubiquitinated proteins. Even though ESCRT proteins appear to be essential for MVEs generation, a second ESCRT-independent pathway has been suggested *via* a tetraspanin-dependent mechanism in which CD81, CD9 and CD63 are key players (Stuffers et al., 2009) (Van-Niel et al., 2011). Tetraspanins are integral membrane proteins in exosomes. Through their interaction with other transmembrane and cytosolic proteins as well as lipids, tetraspanins participate in protein loading into exosomes (Villarroya-Beltri et al., 2014).

Once generated, MVBs could undergoes different fates. While some MVBs can fuse with lysosomes to be degraded overlapping with the autophagy pathway, the presence of specific receptors, such as EGFR, direct the MVBs through the recycling endosome allowing the recycle of the receptor back to the plasma membrane. Another fates for the MVBs is the release into the extracellular compartment *via* the secretory pathway (Baixauli et al., 2014) (Fader and Colombo, 2009) (Hassanpour et al., 2020).

Both pathways are regulated by small GTPases of the Rab family. For example, while Rab7 has been involved in the degradation pathway, Rab27 and Rab11 regulate the MVBs fusion to the plasma membrane (Ostrowski et al., 2010).

In contrast to the above described, particles with a size ranging between 100 and 1000 nm are categorized as microvesicles. While exosomes are generated by inward budding, microvesicles are formed by outward budding of cytoplasmic protrusions followed by extracellular membrane scission. In addition, the biosynthesis of MVs is dependent on the interaction between phospholipids and protein of the cytoskeleton (Clancy et al., 2021).

The larger subtype of EVs, with a size up to 5 μm are the apoptotic bodies. The formation of these particles is associated with programmed cell death and the process is characterized by plasma membrane blebbing (more information regarding this process are available in (Raposo and Stoorvogel, 2013).

The size variety of EVs, the heterogeneity and the lack of well identified markers, as well as of standard isolation protocols generated some confusion and debate in the exosome research field. In order to shed some light, in 2014, the International Society for Extracellular Vesicles provided biochemical, biophysical and functional guidelines that should be applied in EVs biology (Lotvall et al., 2014). With the same objective, in the last years, multiple consortiums (EV-TRACK Consortium–2017) and databases (Vesiclepedia–Exocarta) were generated. Given the increasing popularity of exosomes, extensive efforts have been done to improve their isolation methods. Ultracentrifugation, filtration and immunoaffinity isolation are only some examples of the possible isolation methods available for EVs isolation. Although no EV isolation method yet exists that can be considered as a gold standard, since residual proteins and/or lipoproteins remains problematic, differential centrifugation has long been regarded as the gold standard technique (Sluijter et al., 2018).

As above mentioned, exosomes are defined by their size and their specific endosome associated protein contents. However, isolation methods based on ultracentrifugation separation rely on a pure size dependent isolation, without taking in account the intracellular origin of the vesicles. In 2016, the group of Kowal (Kowal et al., 2016) demonstrated that the presence of specific tetraspanins (CD9, CD63 and CD81) characterizes endosome-derived vesicles (exosome) and a couple of years later, in 2019, Jeppesen et al. performed a deep re-assessment of exosome composition generating the background for a clearer understanding of small EVs heterogeneity (Jeppesen et al., 2019). Nevertheless, before these milestone studies, only few reports studying the role of EVs in multiple pathology clearly demonstrated the tetraspanins positivity of the ultracentrifuge derived small vesicles EVs. Since current isolation protocols result in an enrichment of vesicles population rather than their complete purification, it is more accurate to refer to purified vesicle as EVs (Sluijter et al., 2018). For these reasons, in this review we will use the general nomenclature small EVs even if differentially state in the original paper.

In recent years, the identification of the cargo of small EVs has been under investigation.

Two main classes of proteins can be distinguished: 1- proteins that constitute small EVs and are independent from the cells of origin; 2- proteins that are dependent on the cell type and on the cellular pathophysiological condition (He C. et al., 2018).

Typical proteins found in small EVs (most frequently identified on Exocarta) are tetraspanins, proteins involved in membrane transport and fusion (annexins and Rab-GTPases), components of the ESCRT machinery (Alix and TSG101) and proteins facilitating protein folding (Hsp70 and Hsp90). In addition to these constitutively proteins, small EVs transport biomolecules that specifically characterize the pathophysiological status of the producing cells were found (Chen et al., 2016) (Shepherd et al., 2021) (Rezaie et al., 2021).

The specific exosome content could be used as markers for multiple pathologies but in the last year small EVs have been shown to play a role in the development of atherosclerosis and diabetic cardiovascular pathology.

In this review we will provide insight into small EVs as mediators of cardiovascular diseases and we will describe their fundamental role as intercellular connection among multiple cell type (cardiac, vasculature, immune cells).

## The Intracardiac “Whisper Game”

Tight balance of intra-intercellular communication is necessary to maintain heart integrity [for more information please refer to review (Martins-Marques et al., 2021)].

Many pathological stimuli are affecting cell types other than cardiomyocyte, which ultimately induces a phenotypic response in cardiomyocyte (Tirziu et al., 2010). For example, fibroblasts play a key role in regulating multiple functions and activity of the cellular component of the heart compartment. In fact, cardiac fibroblasts modulate cardiomyocyte hypertrophy, contractility and electrical behavior (LaFramboise et al., 2007).

In this context, the evaluation of the miRNA content of small EVs derived from cardiac fibroblast revealed high abundance of many miRNA passenger strands, which normally undergo intracellular degradation, in particular miRNA21–3p (miRNA-21*). This miRNA induces cardiomyocyte hypertrophy by targeting multiple pro-hypertrophic gene expression (Bang et al., 2014).

Fibroblast activation with Angiotensin II (AngII) or TGFβ induces the release of small EVs that, once internalized into endothelial cells or cardiomyocytes, are able to strongly modulate cell behavior (Ranjan et al., 2021).

For example, endothelial cells treated with small EVs derived from TGFβ activated fibroblast displayed impaired functions, characterized by decreased tube formation and cell migration (effects mediated by miRNA200a-3p) (Ranjan et al., 2021). This effect could be part of a signaling cascade where endothelial cells, under stress conditions such as high glucose, activate fibroblast through TGF-β1 enriched small EVs (Zhang et al., 2021) and this activation leads to the release of small EVs loaded with miRNA200a-3p that impairs endothelial functions.

As aforementioned, fibroblasts do not exclusively communicate with endothelial cells but are also able to modulate cardiomyocyte functions.

AngII, a well know hypertrophic stimulus, enhances fibroblast small EVs release through the activation of the AngII receptor types I and II. Treatment of cardiomyocytes with these small EVs upregulates RAS *via* MAPKs and Akt inducing hypertrophy (Lyu et al., 2015).

A similar effect has been shown with small EVs derived from fibroblast treated with TGFβ. These small EVs are able to induce a heart failure phenotype in cardiomyocyte indicating that exosome signaling from fibroblast contributes to disease progression in heart failure (Basma et al., 2019). This communication between fibroblast and cardiomyocyte is not unidirectional. It has been shown that cardiomyocyte derived small EVs are able to induce fibroblast proliferation and differentiation into myofibroblast by transferring miRNA208a (Yang et al., 2018). Moreover, circulating exosomes induced by cardiac pressure overload contain functional angiotensin II type 1 receptors (AT1R). Exogenously administered AT1R-enriched exosomes target cardiomyocytes, skeletal myocytes, and mesenteric resistance vessels and are sufficient to confer blood pressure responsiveness to angiotensin II infusion in AT1R knockout mice (Pironti et al., 2015).

For the purpose of illustration, we can compare these multiple interactions to a symphonic orchestra. As in an orchestra, all the elements need to be properly connected and working in synchro to create a perfect harmony; that, in our case, is a functional heart. When external factors influence one single component, all the others are also affected. In the next chapter we will analyze how small EVs connect different cellular type in multiple cardiovascular diseases.

## Trick and Treat: Small EVs in Diabetes

Diabetes is a complex disorder characterized by a persistent elevated blood glucose level from insulin deficiency or resistance that leads to the development of life-threatening complications. On the basis of the pathophysiology, it is possible to classify diabetes in different subtypes (Wagner et al., 2021). For the purpose of our review, we will mainly focus on Type 1 and Type 2 diabetes.

### Type I Diabetes

**FIGURE 1:**
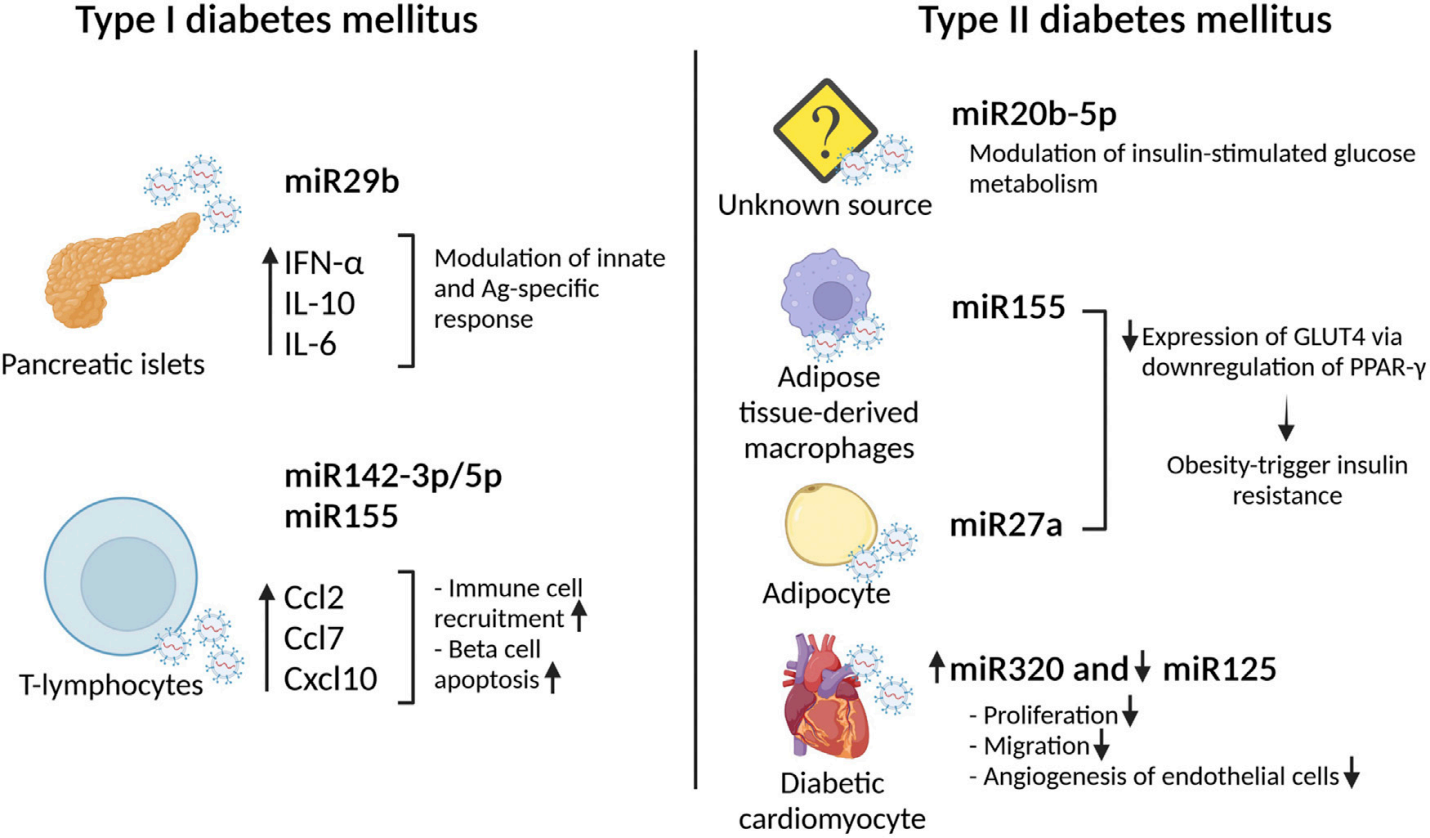
Small EVs miRNA effect in type I and type II diabetes mellitus. Effects of different small EVs loaded with specific miRNA in the development of diabetes.

Type 1 diabetes mellitus (T1DM) is an autoimmune disorder characterized by beta-cell dysfunction leading to insulin deficiency. In the last years, small EVs have been associated to T1DM, in particular in the onset and progression of the disease. Interestingly, it has been shown that small EVs derived from pancreatic islets (human and rat) and mouse insulinoma present disease-specific content such as the island antigens glutamate decarboxylase (GAD65) and islet-associated protein (IA-2) (Sheng et al., 2011), (Cianciaruso et al., 2017). These proteins are able to activate antigen presentation by dendritic cells leading to T-cell activation and the initiation of the autoimmune response (Cianciaruso et al., 2017). If administered *in vivo*, these small EVs are able to induce insulitis in non-obese diabetes resistant mouse models indicating their autoimmunity potential to trigger islets inflammation. Together with auto antigens like GAD65, small EVs derived from pancreatic islets contain distinctive miRNA, such as miRNA29b. This specific miRNA modulates innate and antigen specific immune response by stimulating IFNα, IL-10 and IL-6 secretion (Salama et al., 2014). Other miRNAs playing a role in promoting immune cells recruitment and exacerbating beta cell apoptosis are miRNA142–3p/5p and miRNA155. These miRNAs promote the expression, exclusively in beta cells, of specific chemokine genes such as *Ccl2*, *Ccl7* and *Cxcl10*. Intriguingly, the sources of these microRNAs are T-lymphocyte small EVs (Guay et al., 2019). These observations pointed out the importance of small EVs transfer as a communication mode between immune and insulin-secreting cells. While the aforementioned studies were performed *in vitro* or in murine model, the analysis of small EVs derived from diabetic patients confirmed the presence of deregulated miRNA involved in T1DM progression. These results demonstrate the feasibility of using small EVs derived miRNA signature as clinical applicable biomarkers of T1DM (Guay et al., 2019). Currently, positivity for multiple autoantibodies is the only available biomarker for T1DM (Verge et al., 1996) (Bingley et al., 2003). Recently, the level of miRNA21–5p contained in serum derived small EVs has been proposed as a promising future T1DM biomarker (Lakhter et al., 2018). Unfortunately, due to its key role in regulating vital pathways, miRNA21 has been found frequently deregulated in multiple pathological conditions, from cancer to cardiovascular diseases (Jenike and Halushka, 2021). In this view, miRNA21 could be seen as a potential therapeutic target instead of a biomarker. In this regard, current therapeutic strategies are mainly focusing on attenuating symptoms *via* damping the inflammation and supporting beta cell functions. Treatments with stem cells used to regenerate beta cells have already been proposed, however, next generation therapeutic tools focus now on the potentiality of small EVs delivery (Babaei and Rezaie, 2021). In this context, the comparison of the regenerative effects of mesenchymal stem cells (MSCs) derived small EVs and MSCs themselves has been tested in a T1DM rat model (Sabry et al., 2020). Serum glucose and plasma insulin level were used as read out for the therapeutic effects and pancreatic tissue regeneration was evaluated *via* histology and expression of specific beta cells genes. In both cases, MSCs derived small EVs showed superior results than MSCs themselves. In a murine streptozocin (STZ)-induced T1DM model, the infusion of exosomes derived from adipose tissue-derived MSCs leads to an increase of pancreatic islet and an increase in regulatory T-cell population. In particular, an amelioration of autoimmune reaction is sufficient to induce an increase in islet and the maintenance of blood glucose level (Nojehdehi et al., 2018). Taken together, small EVs could be a potent tool to modulate immune response and thus improve islet functionality and transplantation (Wen et al., 2016) (Figure 1)

### Type 2 Diabetes

Type 2 diabetes (T2DM) is a metabolic disease, occurring mainly in individual over age 45, characterized by insulin resistance largely associated with adiposity and/or by impaired insulin secretion (Jeong et al., 2010). It has been shown that diabetic patients present a greater number of circulating small EVs when compared to normal glucose healthy donor. These specific EVs are mainly generated in response to insulin resistance. Once released, these insulin related EVs, are predominately up-taken by leukocyte and mediate the release of inflammatory cytokine inducing an inflammatory status (Freeman et al., 2018). Another factor playing a pivotal role in T2DM onset and progression is obesity. Also in this case, small EVs may play a role. Indeed, enrichment of specific miRNA has been observed in plasma EVs isolated from obese mice. In addition, administration of these specific EVs in lean mice was able to induce glucose intolerance and insulin resistance, indicating the strict bound between obesity induced release of small EVs and T2DM onset (Castano et al., 2018). From a molecular point of view, *via* binding to the insulin receptor, insulin induces a signaling cascade that involves Akt phosphorylation on position S473 and in turn GLUT4 transporter translocation from the cytosol to the plasma membrane allowing glucose uptake in skeletal muscle cells. Small EVs circulating in T2DM patient’s blood present high levels of miRNA20b-5p that modulate insulin-stimulated glucose metabolism by regulating Akt signaling pathway (Katayama et al., 2019). While the origins of the aforementioned EVs are yet unknown, it has been demonstrated that EVs containing miRNA155 or miRNA27a, originated from adipose tissue-derived macrophages and adipocytes respectively, are able to decrease the expression of GLUT4 *via* downregulation of PPARγ playing thus a role in the development of obesity-triggered insulin resistance (Ying et al., 2017) (Yu et al., 2018) Figure 1.

Multiple pathological conditions characterizing diabetes such as high levels of systemic inflammatory cytokines and chronic hyperglycemia are consider risk factors for cardiac damage. In particular, the deleterious effect of persistent high glucose levels leads to cardiac dysfunction known as diabetic cardiomyopathy (DCM). In this context, diabetic hearts display defective angiogenesis. Co-culture experiments of cardiac endothelial cells with diabetic cardiomyocyte provided evidence that the cardiomyocytes modulate specific endothelial functions such as proliferation inhibition (Wang et al., 2014). Further analysis demonstrated that small EVs derived from diabetic cardiomyocyte inhibit proliferation, migration and angiogenesis capabilities of endothelial cells (Wang et al., 2014). In particular, these EVs presented high levels of miRNA320 and low levels of miRNA125 in comparison to EVs derived from non-diabetic cardiomyocytes *in vivo*. Downregulation of miRNA320 targets including IGF-1, Hsp20 and Ets2 inhibited endothelial migration and tube formation indicating that EVs derived from cardiomyocytes exert an anti-angiogenic function upon T2DM (Wang et al., 2009). Interestingly, one of the miRNA320 targets, Hsp20 has been involved in the development of DCM. Cardiac specific Hsp20 overexpression induces the release of EVs able to attenuate cardiac disfunctions. Of importance, these EVs present higher levels of p-Akt, Survivin, and SOD1, molecules able to promote endothelial cell proliferation and to protect against cardiac adverse remodeling (Wang et al., 2016). Microvascular rarefaction guided by endothelial dysfunction is one of the major signs of DCM, thus, the maintenance of functional/vital endothelial compartment also leads to cardiomyocyte survival and efficient cardiac functions (Kibel et al., 2017) (Figure 1).

### Relevance of Exercise for EVs in T2DM

**FIGURE 2:**
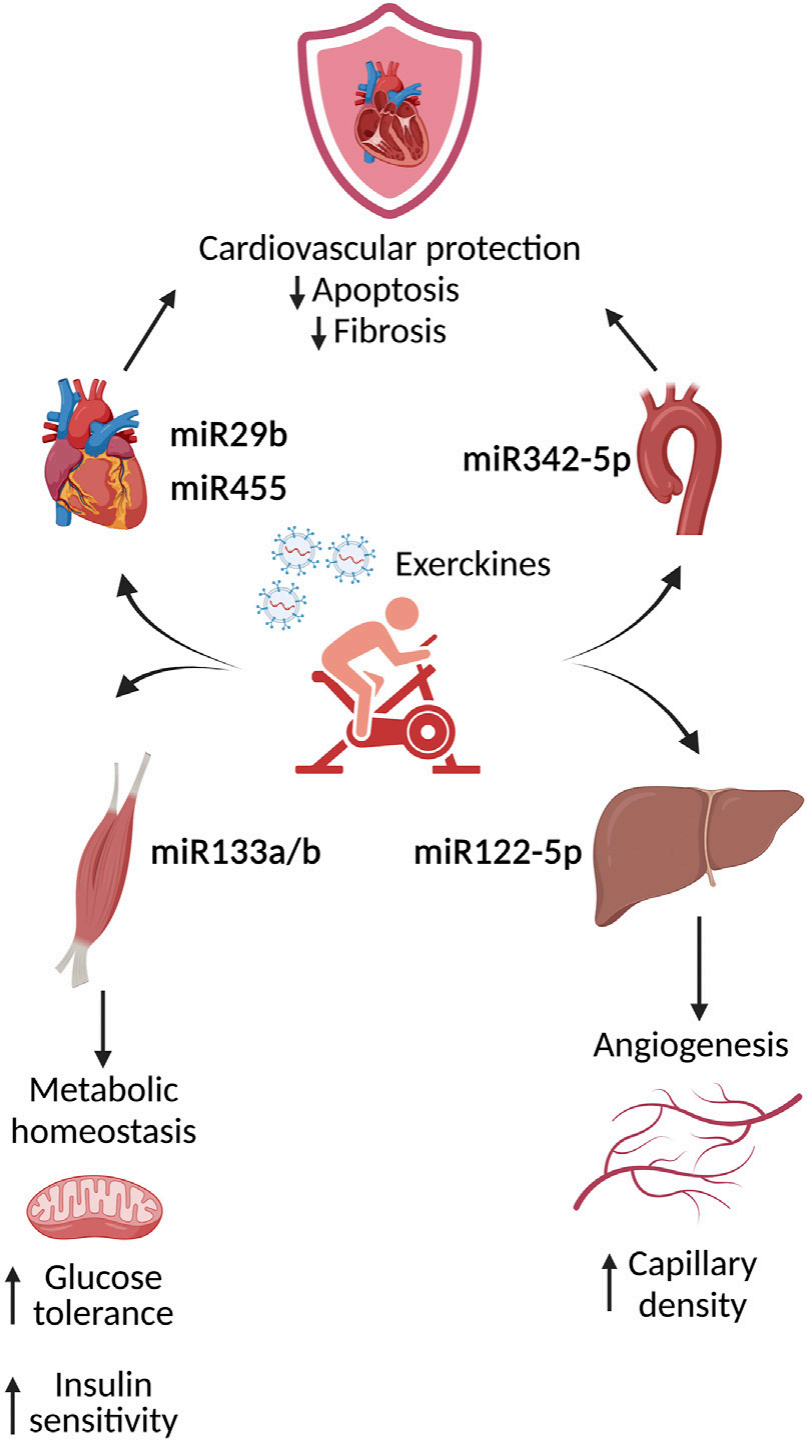
Physiological effects of the induced exerkines upon exercise. During exercise, multiple organs release exosome containing factors, such as miRNA, called exerkines. These molecules are able to activate intra-intercellular pathways leading to an amelioration and protection of the cardiovascular compartment.

One interesting preventive/therapeutic approach for T2DM is the implementation of a regular physical activity. In fact, multiple studies show that diabetic cardiovascular complications benefit from exercise. Endurance exercise induces the release, *via* small EVs, of factors named “exerkines” able to potentially counteract diabetic effects and thus representing a potential therapeutic approach to the treatment of obesity and associated disorders (Safdar and Tarnopolsky, 2018). In particular, physical activity is associated with a significant increase of small EVs circulating in the blood of healthy individuals subjected to an exercise protocol (Fruhbeis et al., 2015). The content of these EVs, including plasma based circulating miRNA, has been involved in angiogenesis, inflammation, cardiac contractility and hypoxia/ischemia adaptation (Baggish et al., 2011). One of the first studies analyzing the effect of EVs in a diabetic context demonstrated that in response to exercise, the release in the heart tissue and serum of small EVs containing specific miRNA (miRNA29b and miRNA455) is able to downregulate MMP9 expression in the heart, thus mitigating cardiac fibrosis. These results lead to the hypothesis that exercise mediated release of small EVs loaded with MMP9 silencing miRNA could represent a potential therapeutic strategy to treat diabetic patients presenting cardiovascular dysfunctions (Chaturvedi et al., 2015). A more recent study demonstrates that, in mice, high intensity interval training induces an increase of circulating EVs released by the muscles, with high expression of miRNA133a and miRNA133b. These miRNAs, targeting the insulin downstream transcription factor FoxO1, are able to induce metabolic effects in the liver such as a reduction in gluconeogenesis. Overall, the iv injection in sedentary mice with these EVs improves glucose tolerance and insulin sensitivity, indicating an improvement of the metabolic homeostasis (Castano et al., 2020). Not only these “exercised” small EVs are able to ameliorate a metabolic impairment, but it has also been shown that small EVs isolated from the plasma of exercised humans or rats, exert a protective effect in an *in vivo* model of myocardial ischemia/reperfusion injury. In particular, these effects were attributed to miRNA342–5p and its antiapoptotic effects in cardiomyocytes. The study of the origin of the exosomal miRNA342–5p highlighted the importance of aortic endothelial cells. In particular, *in vitro* studies determined that fluid shear stress induces miRNA342–5p expression and its subsequent release from endothelial cells. These data indicate that the increase blood flow/shear stress triggered by exercise could potentially confer salutary cardioprotective effects by inducing the generation of exerkines (Hou et al., 2019). Another of the beneficial effects of exercise is the promotion of angiogenesis (Laufs et al., 2004). In this view, it has been shown that exercise could induce the release from the liver of small EVs loaded with miRNA122–5p that promote an increase of capillary density in skeletal muscles (Lou et al., 2021). On the basis of what we describe here, multiple organs benefit from exercise and these effects are modulated by small EVs release (Figure 2). For a comprehensive analysis of the role of endurance exercise and small EVs in the treatment of metabolic disorder we suggest the following exhaustive reviews (Safdar et al., 2016) (Safdar and Tarnopolsky, 2018).

## Small EVs in Atherosclerosis: The Blueprint of the Wall

Atherosclerosis is a complex multifactorial degenerative disease involving metabolism, the cardiovascular and the immune systems. Endothelial and immune cells activation due to the exposure to cardiovascular risk factor such as obesity, smoking and hypertension, and the subsequent foam cells accumulation, are events required for the formation of the atherosclerotic plaque (Mudau et al., 2012). Exchange of information between these cellular types is a fundamental driver of the pathology (Roy et al., 2021). In support of this, in the last years, small EVs have been shown to be involved in multiple steps of pathological progression of atherogenesis.

Endothelial cell inflammation is the initial step driving the cascade of events leading to the atherosclerotic plaques. After endothelial cells activation, cells from the immune compartment, especially monocytes, are recruited and adhere to the arterial lumen where they form the plaque (for a comprehensive analysis regarding the role of macrophages in atherogenesis please refer to (Farahi et al., 2021). Small EVs released by monocytes stressed by oxidized lipoprotein (oxLDL) are able to induce pro adhesion molecules, the NO pathway and to exacerbate vascular inflammation (Madrigal-Matute et al., 2015), (Mastronardi et al., 2011), (Tang et al., 2016).

**FIGURE 3:**
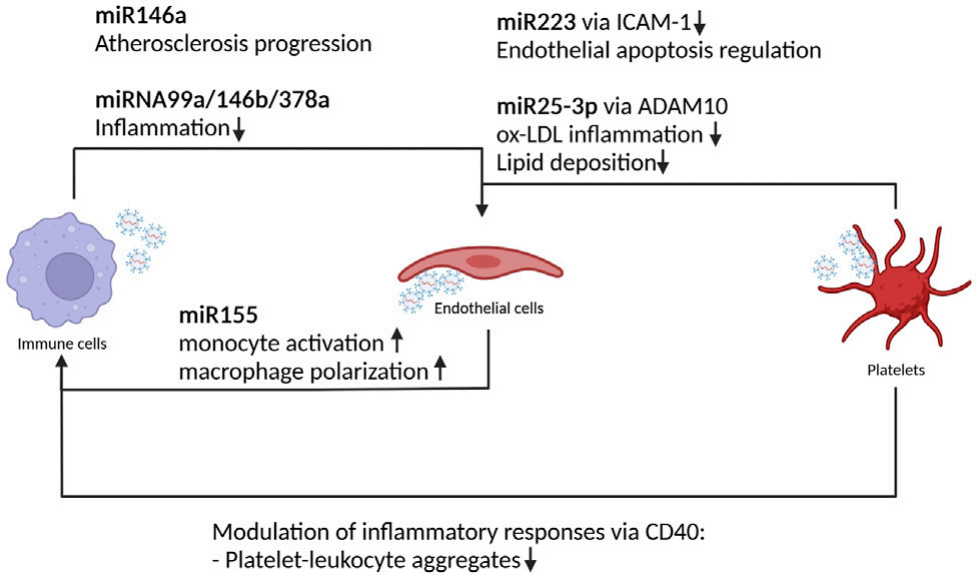
Platelets, immune and endothelial cells derived EVs miRNA effect in atherosclerosis. Schematic representation of the crosstalk between immune cells, EC and platelets driving atherosclerosis pathogenesis. The exchange of miRNA modulates inflammatory response and endothelial activation status leading to atherosclerosis onset and progression.

The tight interconnection between endothelial cells and macrophages has been investigated. While small EVs derived from oxLDL-treated endothelial cells promote M2 macrophage polarization, (Huang et al., 2018), small EVs released from macrophages negatively regulate endothelial cell migration stimulating the trafficking of internalized β1 integrin to the lysosomal compartment resulting in proteolytic degradation (Lee et al., 2014). A peculiarity of small EVs derived from atherogenic macrophages is the capability of the transfer of specific miRNA, e.g., miRNA146a, and thus inducing an atherogenic phenotype. In particular, the treatment of naïve macrophages with small EVs derived from atherogenic macrophages is able to inhibit their migration at the same extend as the donor macrophages (Nguyen et al., 2018). A follow up study on exosomal miRNA146a secreted by oxLDL-treated macrophages demonstrated the role of this specific miRNA in promoting ROS and NETs release *via* targeting SOD2. In particular, miRNA146a induces oxidative stress promoting NETs formation and leading to atherosclerosis progression (Zhang et al., 2019). Furthermore, it has been shown that small EVs produced by polarized (IL4) bone marrow-derived macrophages contain anti-inflammatory miRNAs (miRNA99a/146b/378a) able to suppress inflammation and foster M2 polarization in recipient macrophages. Repeated infusions of these particular small EVs into ApoE^−/−^mice [the most widely used preclinical model of atherosclerosis (Jawien, 2012)] fed a Western diet lead to a reduction in necrotic lesion areas. This collectively stabilizes atheroma indicating that small EVs produced by cultured macrophages have an anti-inflammatory effect. Furthermore, polarized macrophages produce exosomes with an enhanced capacity to resolve inflammation *via* anti-inflammatory miRNA cargo, including in atherosclerotic lesions (Bouchareychas et al., 2020) (Figure 3).

As aforementioned, small EVs derived from cells of the immune compartment are able to modulate the activation status of endothelial cells. Is this true also in the other direction? It has been shown that small EVs released from TNFα stimulated endothelial cells are loaded with a cocktail of inflammatory markers, chemokines, and cytokines (such as ICAM-1, CCL-2, IL-6, IL-8, CXCL-10, CCL-5, and TNF-α) which, when transferred to monocyte are able to induce reprogramming toward a pro- or anti-inflammatory phenotypes (Hosseinkhani et al., 2018). Another study demonstrated that macrophage treatment with small EVs derived from ox-LDL stimulated endothelial cells are able to modulate monocyte/macrophage phenotype. In particular, these small EVs are rich of miRNA155. This particular miRNA stimulates monocyte activation and promotes macrophage polarization towards proinflammatory M1 macrophages. The authors were able to reverse this phenotype taking advantage of the properties of the Kruppel like factor 2 (KLF2) (He S. et al., 2018). KLF2 is a shear stress induced transcriptional factor with protecting effects against atherosclerosis. In particular, KLF2 modulates the expression of different miRNA cluster (such as the miRNA143/145). *In vivo*, small EVs from KLF2-expressing endothelial cells reduced atherosclerotic lesion formation in the aorta of ApoE ^−/−^ mice (Hergenreider et al., 2012). They also suppressed monocyte activation by enhancing immunomodulatory responses and diminishing proinflammatory responses by decreasing proinflammatory M1 macrophages and increasing anti-inflammatory M2 macrophages (He S. et al., 2018).

In an already complex cellular network leading to plaque formation, the role of a new player has been recently pinpointed, namely the platelets (Hundelshausen and Lievens, 2011). Platelet-derived small EVs modulate multiple endothelial and monocyte functions (Barry et al., 1998). In particular, small EVs from thrombin activated platelet modulate inflammatory immune responses *via* cluster of differentiation 40 (CD40) ligands (Kaneider et al., 2003). Platelet CD40 mediates the formation of platelet-leukocyte aggregates and, in absence of CD40, leukocytes are less prone to adhere to thrombi. In an ApoE^−/-^ mouse model, plaques of mice receiving CD40-deficient platelets are less advanced and contain less immune cell infiltration (Gerdes et al., 2016). Thrombin activated platelet are enriched with miRNA223 that inhibits ICAM-1 expression during inflammation and regulates endothelial apoptosis (Li et al., 2017). Another miRNA involved in atherosclerosis and highly expressed in platelet small EVs is miRNAR25–3p that, when acting on the ADAM10 metalloprotease, inhibits ox-LDL inflammation and lipid deposition (Yao et al., 2019). Figure 3.

Through an organ culture approach, the *in vitro* analysis of the inflammatory process occurring in human atherosclerosis demonstrated that the monocyte-platelet interaction is driving the composition of specific small EVs. In particular, the platelet’s activation status plays a pivotal role in the small EVs pro inflammatory content. Inhibition of platelets activation leads to a milder inflammatory phenotype, suggesting a synergistic role of monocyte/platelet aggregates in releasing functional EVs (Oggero et al., 2021).

## Summary/Conclusion

Taken together, we can conclude that the EVs mediated signaling adds a higher complexity to an already complex mechanism of direct cell-to-cell interaction, such as monocyte-endothelial cell. The discovery of small EVs is allowing to understand new types of cellular communication and at the same time could become a powerful tool for the development of new therapeutic approaches. Besides the therapeutic use of EVs in cardiovascular disease as a scavenger for e.g., miRNAs, they might also be suitable for novel biomarkers allowing for a precise and early disease diagnosis, as well as prognostic marker for the progression of the cardiovascular disease.
